# Interstitial pneumonia as the initial presentation in an infant with a novel mutation of CD40 ligand-associated X-linked hyper-IgM syndrome: A case report

**DOI:** 10.1097/MD.0000000000020505

**Published:** 2020-06-12

**Authors:** Jian Li, Hongjun Miao, Lihui Wu, Yongjun Fang

**Affiliations:** a Department of Hematology Oncology; b Department of Emergency Medicine, Children's Hospital of Nanjing Medical University, Nanjing, PR China.

**Keywords:** CD40 ligand deficiency, cytomegalovirus, Hyper-IgM syndrome, infant, *Pneumocystis jirovecii*

## Abstract

**Introduction:**

X-linked hyper-IgM syndrome is a type of primary combined immunodeficiency disorder caused by mutations in CD40 ligand. Opportunistic infections caused by *P jirovecii*, cytomegalovirus (CMV), or fungi are frequently the first presenting symptom of the patients with X-linked hyper-IgM syndrome.

**Patient concerns:**

Here, we report a 10-month-old infant who presented with cyanosis and shortness of breath. The infant exhibited no medical or birth history indicating a primary immune deficiency and was first diagnosed with interstitial pneumonia and acute respiratory failure on admission.

**Diagnoses:**

The infant was diagnosed with *Pneumocystis jirovecii* pneumonia combined with CMV and fungal infection through gene sequencing by nasopharyngeal swab and G-test. Whole-exome sequencing from a blood sample was performed and identified a functional mutation across the CD40 ligand gene (NM_000074;exon1;C.86_87del) resulting in an amino acid change (P.T29Sfl∗18) attributed to X-linked hyper IgM syndrome.

**Interventions:**

The infant received continuous positive airway pressure ventilation treatment combined with trimethoprim-sulfamethoxazole for *Pneumocystis jirovecii* pneumonia, ganciclovir for CMV, voriconazole for fungal infection and substitution of high-dose immunoglobulin.

**Outcomes:**

Six months after discharge from our hospital, the infant remained well.

**Conclusion:**

Opportunistic infections should be suspected in infants presenting with severe interstitial pneumonia. Primary immune deficiency diseases should also be considered in infants diagnosed with opportunistic infections.

## 1 Introduction

X-linked hyper-IgM syndrome (XHIGM) caused by the CD40 ligand (CD40L) mutation is a rare primary immunodeficiency condition that decreases immunity to opportunistic infections because there are defects in neutrophils, complement proteins, macrophages, and lymphocyte subsets.^[1,2]^ A missed or delayed diagnosis of XHIGM is widespread and concerning, especially in patients without a relevant family or medical history. Distinct clinical infectious complications could occur in those with XHIGM syndrome based on the genetic characteristics and the exact type of the syndrome, which makes it a challenge for researchers and clinicians. The respiratory tract was the most commonly involved organ in XHIGM patients, which also included the gastrointestinal tract, skin, central nervous system, and septicemia. The most common opportunistic pathogens include *Pneumo-cystis pneumonia* (PCP), fungal infection, *Cryptosporidium* infection, and infections by members of the herpesvirus family.^[1,3–5]^

Here, we report a male infant with a novel mutation of CD40L who developed severe interstitial pneumonia and respiratory failure with PCP, cytomegalovirus (CMV) and fungal infection.

### 1.1 Case presentation

**Figure 1:**
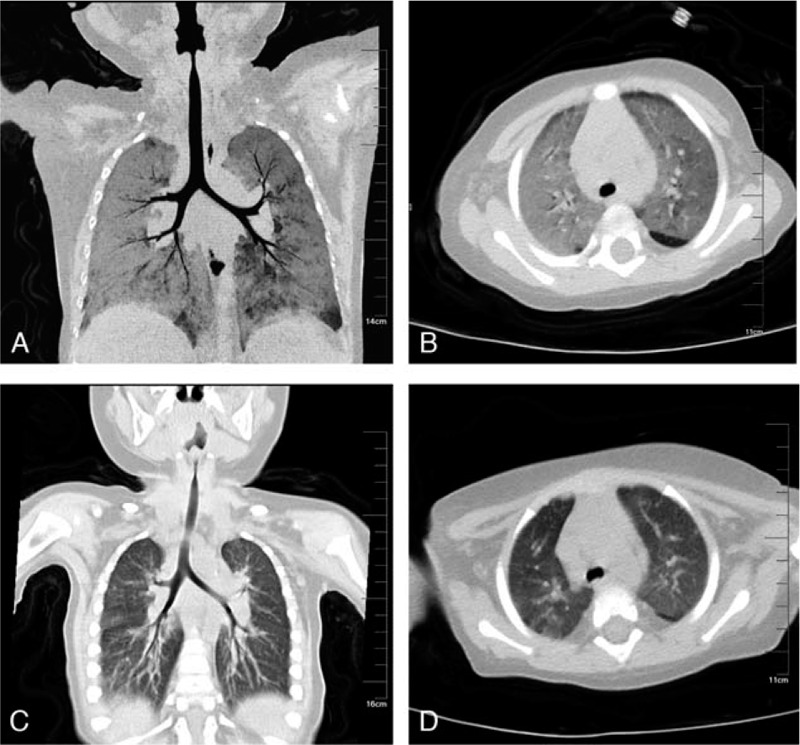
Chest CT before (A/B) and after (C/D) treatment. CT = computed tomography.

A 10-month-old boy with recurrent fever, cough, tachypnea and cyanosis for 8 days with a history of antibiotic therapy was admitted to our emergency department with suspected pneumonia. This infant was the only child of his family and exhibited no medical problems at or after birth. Parents of this infant were both healthy. On admission, the infant respiratory status worsened and required ventilatory support. Blood gas analysis showed the following: pH 7.35; PCO2 32 mmHg; PO2: 71.3 mmHg; BE: -7.0, HCO3: 18.9 mmol/L; and Lac 1.5. Routine blood analysis showed white blood cell (WBC) 11; 1000 mm with cell differentiation segment: 9.1%; lymphocyte: 81.6%; eosinophil: 5.2%; platelet: 288 mm 1000 mm; and Hb: 10.7 g/dL. C-reactive protein was evaluated using serological test and showed 8 mg/L. Chest computed tomography can showed diffuse nonsegmental ground glass opacity in both lungs with left axillary lymph node calcification [Fig. 1]. To maintain the oxygen supply of the infant, we used noninvasive ventilation. Pulse oximetry showed an SpO2 of 80% in room air, which rose to 98% with oxygen supplied at 5 L/min. The disease is severe and progresses rapidly. Thus, the standard procedure in our department for severe respiratory infection was immediately started on day 2, which comprises extended pathogen screening, an immunological check and markers of inborn errors of metabolism.

The infant^,^s immunologic evaluation revealed low IgG (<1.39 g/L, normal: 4.09 -7.03 g/L), low IgA (<0.0647 g/L, normal: 0.21–0.43 g/L), and high levels of IgM (1.89 g/L, normal: 0.33–0.73 g/L). Complement proteins C3 and C4 were 0.912 g/L and 0.167 g/L, respectively. Blood and urine genetic metabolism screening was normal. Serologic investigations for common viruses were normal. The PPD test for tuberculosis was negative. IgG and IgM against mycoplasma were evaluated using ELISA and were negative. Sputum and blood culture examination was likewise normal. EBV and CMV-DNA analyses were performed on a blood sample and were negative. A multiplex polymerase chain reaction (PCR) test for respiratory viruses was performed using a nasopharyngeal swab and showed positive CMV-LRB-2.60E+8 copies. The G-test was performed and was positive (152.4 pg/mL), while the GM-test was normal. For further analysis, next-generation sequencing (NGS) for nasopharyngeal swabs was performed, and the results can be found in Table 1. Thus, the diagnosis of pneumonia from PCP combined with CMV and fungal infection was made.

**Table 1:**
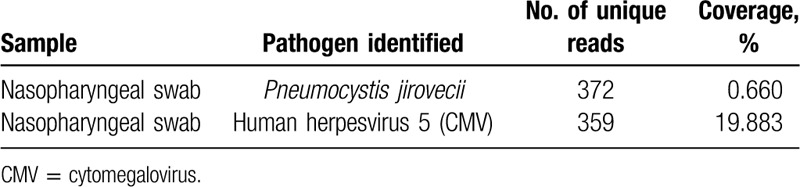
Next-generation sequencing results of the main pathogens in the nasopharyngeal swab sample.

Due to the abnormal status of immunoglobulin levels, XHIGM syndrome was suspected, and this hypothesis was subsequently confirmed. Genetic analysis performed through whole-exome sequencing (WES) and Sanger sequencing of CD40LG revealed amino acid changes in exon 1 of CD40LG (C.86_87del). The above mutations were analyzed by protein function prediction software REVEL (rare exome variant ensemble learner) and predicted to be pathogenic mutations, thus confirming the diagnosis of XHIGM (Table 2). Family DNA analysis indicated that neither of his parents carried the mutation found in the infant; thus, this mutation is a novel de novo spontaneous deletion mutation that was not previously reported.

**Table 2:**
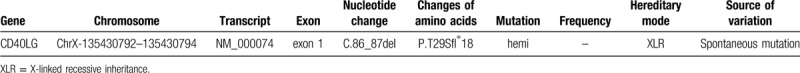
DNA variation in the patient. The mutations were not reported in the human genome mutation database. MutationTaster software (www.mutationtaster.org) was used to predict the pathogenicity of the mutations.

Thus far, the diagnosis is quite clear. The infant was treated with substitution of high-dose immunoglobulin (2 g/kg) and methylprednisolone (2 mg/kg iv) to reduce the inflammation reaction. Meropenem was adminstered to prevent bacterial infection. Trimethoprim-sulfamethoxazole was utilized to treat PCP. CMV infection was treated with ganciclovir. Fungal infection was treated with voriconazole. The clinical manifestations of shortness of breath were gradually relieved, and continuous positive airway pressure ventilation was stopped after 3 weeks. Quantification of CMV DNA showed a gradual decline *(*2.60E+8 copies to 1.11E+5 copies*).* A repeat chest CT showed that pneumonia gradually improved (Fig. 1). The infant healed and was discharged from the hospital after an overall time of 4 weeks. Now, the infant is regularly treated with immunoglobulin replacement and is under continued antibiotic prophylaxis with trimethoprim-sulfamethoxazole. There was no further episode of significant infection, and the infant remained asymptomatic during the course of the next 6 months of follow-up.

## 2 Discussion

X-linked hyper-IgM syndrome (XHIGM) is a combined immunodeficiency caused by mutations in CD40LG, which results in an inability to signal B cells to undergo isotype switching; thus, B cells produce only IgM and lead to elevated IgM levels and absent IgG, IgA, and IgE. The humoral immunity results shown in this infant indicate that X-linked hyper IgM syndrome is the underlying disorder. We performed whole-exome sequencing (WES), and the details are described in Table 1. A nucleotide deletion at cDNA position C.86_87 in the CD40L gene is predicted to change the reading frames and lead to large changes in the protein structure.

HIGM can be divided into seven subtypes according to gene defects. XHIGM, which is caused by CD40L mutations, is the most common form of HIGM and covers approximately 65% to 70% of all cases.^[2,6]^ Mutations of the CD40L gene are highly heterogeneous. Most CD40L gene mutations are missense mutations, and more than 160 mutations have been recorded thus far.^[1,6]^ More than 20 genetic mutations have been described in Chinese children.^[7]^ Mutation hotspots are mostly concentrated in exon 5. In this study, a rare deletion mutation located in exon 1 was found and analyzed by using MutationTaster software to predict the pathogenicity of the mutation. This variant has not been reported previously and was confirmed by Sanger sequencing.

Patients with XHIGM usually develop symptoms in the first or second year of life, which is in accordance with our study. The most prominent clinical feature observed in XHIGM is respiratory tract infections due to common bacterial pathogens as well as opportunistic infections and leads to high mortality. Opportunistic infections caused by *P. jirovecii* (PCP) are frequently the first presenting illness in male infants with XHIGM.^[1,4]^ They also have an increased susceptibility to a wide variety of infectious agents, including *Cryptosporidium*, cytomegalovirus (CMV), fungi, and parasites.^[4,8,9]^ In this case, we used NGS for nasopharyngeal swabs and finally demonstrated the mixed infection of PCP combined with CMV and fungal infection, which is consistent with previous research results. Over 50% of patients have chronic or intermittent neutropenia, often associated with oral ulcers, which were also found in our case. In addition to the above clinical manifestations, other involvements, such as liver disease, arthritis, anemia, inflammatory bowel disease and neoplasms, may indicate this disease. Patients who presented with pulmonary alveolar proteinosis and severe cutaneous histoplasmosis were also reported recently.^[10,11]^

Children with XHIGM often need regular infusion of human immunoglobulin replacement therapy to prevent *Pneumocystis carinii* infection, actively treat *Cryptosporidium* infection, and choose hematopoietic stem cell transplantation treatment according to need. In this case, the infant was treated with trimethoprim-sulfamethoxazole for PCP, ganciclovir for CMV and voriconazole for fungal infection. The infant also received a substitution of high-dose immunoglobulin. To date, 6 months have passed after discharge from the hospital without severe complications. This finding shows us that the mortality rate of XHIGM can decrease if the disease can be diagnosed early, its management is regulated, and serious infections are avoided.

In conclusion, we report a 10-month-old male infant who presented with severe interstitial pneumonia and was diagnosed with PCP combined with CMV and fungal infection as the initial manifestation of underlying XHIGM syndrome. Moreover, a novel mutation in exon 1 of the CD40L gene was also found for the first time in this study. Early detection and diagnosis are necessary for children with XHIGM, especially in those with opportunistic infections and no relevant family or medical history patients.

## Author contributions

**Data curation:** Lihui Wu, Jian Li.

**Funding acquisition:** Yongjun Fang.

**Project administration:** Lihui Wu.

**Resources:** Hongjun Miao, Yongjun Fang.

**Writing – original draft:** Jian Li.

**Writing – review & editing:** Yongjun Fang.
